# Hepatocellular Carcinoma Is a Natural Target for Adeno-Associated Virus (AAV) 2 Vectors

**DOI:** 10.3390/cancers14020427

**Published:** 2022-01-15

**Authors:** Nadja Meumann, Christian Schmithals, Leroy Elenschneider, Tanja Hansen, Asha Balakrishnan, Qingluan Hu, Sebastian Hook, Jessica Schmitz, Jan Hinrich Bräsen, Ann-Christin Franke, Olaniyi Olarewaju, Christina Brandenberger, Steven R. Talbot, Josef Fangmann, Ulrich T. Hacker, Margarete Odenthal, Michael Ott, Albrecht Piiper, Hildegard Büning

**Affiliations:** 1 Institute of Experimental Hematology, Hannover Medical School, 30625 Hannover, Germany; Meumann.Nadja@mh-hannover.de (N.M.); Franke.Ann-Christin@mh-hannover.de (A.-C.F.); Olarewaju.Olaniyi@mh-hannover.de (O.O.); ulrich.hacker@medizin.uni-leipzig.de (U.T.H.); 2 REBIRTH Research Center for Translational Regenerative Medicine, Hannover Medical School, 30625 Hannover, Germany; 3 Center for Molecular Medicine Cologne, University of Cologne, 50931 Cologne, Germany; m.odenthal@uni-koeln.de; 4 Department of Medicine 1, University Hospital, Goethe University Frankfurt, 60590 Frankfurt, Germany; Christian.Schmithals@kgu.de (C.S.); Piiper@med.uni-frankfurt.de (A.P.); 5 Fraunhofer Institute for Toxicology and Experimental Medicine Preclinical Pharmacology and In-Vitro Toxicology, 30625 Hannover, Germany; leroy.elenschneider@item.fraunhofer.de (L.E.); tanja.hansen@item.fraunhofer.de (T.H.); 6 Clinic for Gastroenterology, Hepatology and Endocrinology, Hannover Medical School, 30625 Hannover, Germany; balakrishnan.asha@mh-hannover.de (A.B.); Hu.Qingluan@mh-hannover.de (Q.H.); Hook.Sebastian@mh-hannover.de (S.H.); Ott.Michael@mh-hannover.de (M.O.); 7 Twincore Centre for Experimental and Clinical Infection Research, 30625 Hannover, Germany; 8 Nephropathology Unit, Institute of Pathology, Hannover Medical School, 30625 Hannover, Germany; Schmitz.Jessica@mh-hannover.de (J.S.); Braesen.Jan@mh-hannover.de (J.H.B.); 9 Institute of Functional and Applied Anatomy, Hannover Medical School, 30625 Hannover, Germany; Brandenberger.Christina@mh-hannover.de; 10 Biomedical Research in Endstage and Obstructive Lung Research (BREATH), German Center for Lung Research (DZL), 30625 Hannover, Germany; 11 Institute for Laboratory Animal Science, Hannover Medical School, 30625 Hannover, Germany; Talbot.Steven@mh-hannover.de; 12 KRH Klinikum Siloah, Liver Center Hannover (LCH), 30459 Hannover, Germany; Josef.Fangmann@krh.eu; 13 Department of Oncology, Gastroenterology, Hepatology, Pulmonology, and Infectious Diseases, University Cancer Center Leipzig (UCCL), Leipzig University Medical Center, 04103 Leipzig, Germany; 14 Institute of Pathology, University Hospital Cologne, 50931 Cologne, Germany; 15 German Cancer Consortium (DKTK), German Cancer Research Center (DKFZ), 69120 Heidelberg, Germany; 16 German Center for Infection Research (DZIF), Partner Site Hannover-Braunschweig, 38124 Braunschweig, Germany

**Keywords:** AAV vectors, hepatocellular carcinoma, liver, cancer gene therapy

## Abstract

**Simple Summary:**

Gene therapy is a novel approach to treat diseases by introducing corrective genetic information into target cells. Adeno-associated virus vectors are the most frequently applied gene delivery tools for in vivo gene therapy and are also studied as part of innovative anticancer strategies. Here, we report on the natural preference of AAV2 vectors for hepatocellular carcinoma (HCC) compared to nonmalignant liver cells in mice and human tissue. This preference in transduction is due to the improved intracellular processing of AAV2 vectors in HCC, resulting in significantly more vector genomes serving as templates for transcription in the cell nucleus. Based on this natural tropism for HCC, novel therapeutic strategies can be designed or existing therapeutic approaches can be strengthened as they currently result in only a minor improvement of the poor prognosis for most liver cancer patients.

**Abstract:**

Although therapeutic options are gradually improving, the overall prognosis for patients with hepatocellular carcinoma (HCC) is still poor. Gene therapy-based strategies are developed to complement the therapeutic armamentarium, both in early and late-stage disease. For efficient delivery of transgenes with antitumor activity, vectors demonstrating preferred tumor tropism are required. Here, we report on the natural tropism of adeno-associated virus (AAV) serotype 2 vectors for HCC. When applied intravenously in transgenic HCC mouse models, similar amounts of vectors were detected in the liver and liver tumor tissue. In contrast, transduction efficiency, as indicated by the level of transgene product, was moderate in the liver but was elevated up to 19-fold in mouse tumor tissue. Preferred transduction of HCC compared to hepatocytes was confirmed in precision-cut liver slices from human patient samples. Our mechanistic studies revealed that this preference is due to the improved intracellular processing of AAV2 vectors in HCC, resulting, for example, in nearly 4-fold more AAV vector episomes that serve as templates for gene transcription. Given this background, AAV2 vectors ought to be considered to strengthen current—or develop novel—strategies for treating HCC.

## 1. Introduction

Liver cancer, including HCC, is the sixth most common cancer worldwide and was the third-highest cause of cancer-related death in 2020, with approximately 906,000 new cases and 830,000 deaths [1]. According to a projection from 30 countries worldwide, numbers will continue to rise to more than 1 million deaths in 2030 [2]. Most HCC occurs in patients with chronic liver disease or liver cirrhosis due to viral infections (i.e., chronic hepatitis B or C) or alcohol abuse. While improved antiviral treatment is expected to decrease the incidence of viral infection-triggered HCC, the incidence of nonalcoholic fatty liver disease (NAFLD)-related HCC is steadily increasing due to the ongoing obesity pandemic in Western countries [3].

The Barcelona Liver Cancer staging classification represents a cornerstone for treatment decisions [4]. Local treatment approaches such as resection, liver transplantation, or chemoembolization play a key role in early disease stages. For advanced disease, multi tyrosine kinase inhibitors (i.e., sorafenib, lenvatinib, regorafenib, cabozantinib) and anti-angiogenic drugs (i.e., bevacizumab, ramucirumab) have demonstrated some efficacy. However, in over 80% of cases with advanced disease, the prognosis has remained poor, with a median overall survival of between 11 and 13 months [5]. More recently, immunotherapy has evolved as a new treatment approach for HCC as the anti-PD-1 antibodies pembrolizumab and nivolumab, as well as a combination of nivolumab plus ipilimumab (anti-CTLA-4 antibody), were approved for second-line treatment, i.e., following progression during tyrosine kinase inhibitor treatment [5]. Finally, improved progression-free survival and overall survival in the first-line treatment of advanced unresectable HCC, compared to sorafenib treatment, was achieved by combining the anti-angiogenic antibody bevacizumab and the anti-PD-L1 immune checkpoint inhibitor atezolizumab, fostering its market authorization by the FDA [6].

Gene therapy is becoming an attractive strategy to complement current treatment strategies and further improve the treatment of cancer diseases [7]. Accordingly, several vector systems and approaches are currently under development in the direction of localized and advanced liver cancer [6]. Amongst these are adeno-associated virus (AAV) vectors. With more than 405 human clinical trials and three market-approved gene therapy drugs, AAV vectors are by far the most frequently used delivery system, particularly for in vivo gene therapy [8,9]. They are applied in the late stages of human clinical trials for the treatment of monogenetic liver-based diseases such as hemophilia B [10]. In addition, AAV vectors are explored in the context of HCC [11,12,13,14,15,16,17,18,19,20,21,22,23,24,25,26]. These studies were predominantly performed in subcutaneous xenograft models using AAV2, AAV8, and AAV3. AAV3, a naturally occurring human AAV serotype, and AAV3-derived engineered capsids (AAV3-Y single and double-mutants, LK03 and AAV3-ST) were reported to show enhanced tropism for human xenograft HCC tissue, with higher efficiency than other AAV serotypes [14,16,27]. This preference is dependent on the presence of the human hepatocyte growth factor receptor (hHGFR) [28], which is overexpressed on the human liver tumor cell surface [29] and explains why AAV3 fails to transduce neighboring murine hepatocytes. AAV3 was reported to efficiently express in nonmalignant human hepatocytes [30,31,32], suggesting that the HCC-tropism observed in human xenograft HCC models is likely a species-related effect.

Here, we report on a thus far unrecognized feature of AAV2. Specifically, when applied to HCC-bearing livers, AAV2 vectors preferentially transduce hepatocellular carcinoma cells. Although most vectorized AAV serotypes accumulate in the liver, transduction efficiency is low considering the number of intravenously (i.v.) or locally applied particles, arguing for the presence of intracellular barriers [10]. We observed that the uptake of AAV2 in hepatocytes and HCC occurs with comparable efficiency. However, intracellular conditions in HCC are more favorable, leading to an up to 19-fold higher efficacy in transduction. This preference was confirmed in different transgenic HCC mouse models in which tumors developed endogenously, induced by overexpression of oncogenes instead of grafted human HCC cells into immune-deficient mice. The same phenomenon was observed in a patient-derived HCC sample, indicating that the preference of AAV2 for HCC transduction is not restricted to mouse models and consequently ought to be considered in the design of future human clinical trials.

## 2. Materials and Methods

### 2.1. AAV Vector Production

AAV2 vectors were produced in HEK293 cells and purified by iodixanol step gradient [33,34]. Genomic particle titers were determined by absolute quantitative polymerase chain reaction (qPCR) using a LightCycler (Roche Diagnostics, Mannheim, Germany) and transgene-specific primers (Table S1).

### 2.2. Animal Studies

Male transgenic transforming growth factor (TGF)α/c-myc bitransgenic mice were bred by crossing homozygous metallothionein/TGFα and albumin/c-myc single transgenic mice in a CD13B6CBA background [35,36]. Hepatocarcinogenesis in the mice was accelerated by adding ZnCl_2_ to the drinking water [50 mM]. TGFα/c-myc mice were monitored for the presence of liver tumors by magnetic resonance imaging (MRI) [36,37]. Animals with tumors of 2.4 to 13.7 mm in diameter (24 to 44 weeks of age) were enrolled. Vector administration was performed within a maximum of one week after the last MRI. For the first in vivo experiment addressing biodistribution and expression levels of sc.AAV2.SFFV.Fluc and sc.AAV2.CMV.eGFP vectors, three subcohorts were analyzed (*N*1 = 3 animals; *N*2 = 3 animals; *N*3 = 2 animals). The subcohorts differed in the date of recruitment and vector administration but were otherwise treated identically. To avoid hydrodynamic injection effects, vectors were injected sequentially. Animals received intravenous (i.v.) administration with 2 × 10^11^ vector particles of scAAV2.SFFV.Fluc on day 0. The same dose of sc.AAV2.CMV.eGFP was administered on day 0 + 12 h. Tissues were isolated 96 h following the first vector administration. Tumor tissue samples were harvested for tissue lysates and DNA isolation as well as histological analysis. In cases where tumors were too small to perform both DNA isolation and a histological analysis, the histological analysis was performed. The same mouse model was used to investigate the transduction pattern in HCC lesions. These tumor-bearing TGFα/c-myc animals (*N* = 6) received 1 × 10^11^ scAAV2.CMV.eGFP vector particles.

In addition, AAV2 expression was monitored in conditional, doxycycline-driven LAP-tTA/TRE (LT2)/RAS and LT2/MYC HCC mouse models [38,39] and in the healthy LT2 and FVB/N wild-type mouse strains. The LT2/MYC mice developed liver tumors around 8–10 weeks following the withdrawal of doxycycline from the diet. The LT2/RAS mice developed HCC 4–6 weeks after doxycycline withdrawal. The LT2 cohort and LT2/RAS cohort differed in the date of recruitment and date of AAV vector administration but were otherwise treated identically. Tumor-bearing animals were i.v. injected with 2 × 10^11^ vector particles of sc.AAV2.SFFV.Fluc. In vivo imaging was performed at the indicated time points after vector injection. Livers were then isolated for in situ imaging.

### 2.3. In Vivo and In Situ Imaging

AAV2 transduction efficiency in the transgenic HCC mouse models LT2/RAS and LT2/MYC (the latter in Figure S1) was analyzed by IVIS^®^ measurements. Animals were anesthetized with isoflurane (5%), received 2.5 mg of D-luciferin potassium salt (Intrace Medical SA, Lausanne, Switzerland) intraperitoneally (i.p.), and were positioned in the IVIS Lumina II in vivo imaging system (PerkinElmer, Baesweiler, Germany). After 10 min, in vivo images were taken with 1 min of exposure. Animals were maintained in isoflurane anesthesia and received another i.p. dose of 2.5-mg D-luciferin. Animals were euthanized after 5 min, and the livers were isolated and in situ imaged (1 or 2 min of exposure). Firefly luciferase activity/luminescence signal was quantified in average radiance (p/s/cm^2^/sr).

### 2.4. Human Precision-Cut Liver Slice (PCLS) Transduction

Human liver and HCC tissue were obtained from patients undergoing partial hepatectomy due to primary or secondary liver cancer. Liver tissue was stored immediately in Belzer UW^®^ Cold Storage solution (Bridge to Life Ltd., London, UK) and processed within 3 h of collection. PCLS were prepared and cultured following the procedure of Granitzny et al. with minor modifications [40]. In brief, cylindrical tissue cores 8 mm in diameter were produced from tissue pieces with a drill equipped with a coring tool and kept in ice-cold Belzer UW^®^ until use. Next, liver slices (Ø 8 mm, 200–300 µm in thickness) were generated in ice-cold Krebs–Henseleit buffer (pH: 7.42) supplemented with 25 mM NaHCO_3_, 25 mM D-glucose, and 10 mM HEPES, and saturated with carbogen (95% O_2_, 5% CO_2_) using a Krumdieck tissue slicer MD6000 (Alabama Research and Development, Munford, AL, USA). Subsequently, PCLS (2 slices/well) were transferred into 24-well plates filled with 1 mL prewarmed and oxygenated William’s E Medium supplemented with GlutaMAX™, 25 mM D-glucose and 50 µg/mL gentamicin (all from Thermo Fisher Scientific, Waltham, MA, USA) and pre-incubated for 2 h at 37 °C, 80 rpm in carbogen-gassed culture boxes within an incubation shaking cabinet Certomat^®^ CT Plus (Sartorius Stedim Systems GmbH, Göttingen, Germany). Thereafter, PCLS were transferred to another 24-well plate filled with either 1 mL Cellartis^®^ Hepatocyte Maintenance Medium (HMM) (Takara Bio Europe SAS, Saint-Germain-en-Laye, France) or Cellartis^®^ HMM containing 4 × 10^10^ scAAV2.SFFV.Fluc vector particles per well (~2 × 10^4^ vg per cell). After 24 h, PCLS were harvested by dividing each PCLS into two parts with a scalpel and shock-frozen in liquid N_2_. PCLS samples were analyzed by luciferase assay and AAV vector DNA quantification. Aliquots of the culture medium were stored at 4 °C and used for the lactate dehydrogenase (LDH) assay.

### 2.5. DNA Isolation and Relative AAV Vector DNA Quantification

Genomic DNA was isolated from shock-frozen tissue samples (10 to 25 mg) by tissue lysis and DNA purification with the QIAGEN DNeasy Blood and Tissue Kit (QIAGEN, Hilden, Germany) according to the manufacturer’s instructions. For determination of AAV vector DNA/genome content, DNA samples were diluted to 20 ng per µL and analyzed by relative qPCR quantification. Values obtained for Fluc transgene were normalized to ng DNA per qPCR reaction (Table S1).

### 2.6. Luciferase Assay

Shock-frozen tissue samples (about 5 to 10 mg) were lysed as described [41] and the protein content was determined by a Bradford assay. Luciferase Assay System (Promega, Mannheim, Germany) was loaded with 20 µL of lysate per sample. Firefly luciferase activity/luminescence signal is shown in relative light units (RLU).

### 2.7. Immunohistochemical Staining and Analysis

Tumor samples, including adjacent liver tissue, were fixed and paraffin-embedded, and 4-µm tissue sections were prepared. IHC staining with anti-eGFP antibody (ab290, Abcam, Cambridge, UK) in combination with the ZytoChem alkaline phosphatase (AP) anti-Rabbit antibody Kit and Fast Red Substrate Pack (Zytomed Systems, Bargteheide, Germany) was performed. Stained tissue sections were digitized by a Leica SCN400 slide scanner (Leica Biosystems, Wetzlar, Germany) with a 20× objective. For analysis of eGFP expression within tumors compared to the adjacent liver (first in vivo experiment), we quantified the relative number of eGFP-positive (red-stained) cells normalized to the tissue area under investigation using Aperio Image Scope Software Version (v12.3.1.5011). For determining the AAV2 transduction pattern within the different layers of the HCC lesions (second in vivo experiment), tissue sections were stained for eGFP as described above. Stained sections were digitized with an AxioScan.Z1 slide scanner (Zeiss, Heidelberg, Germany) at 20× objective magnification. Random images covering 30% of the total area of each tumor section were generated using the Visiopharm software (Version 5.3.1, Visiopharm, Hoersholm, Denmark). We used a random image grid to define the different tumor layers (border, first layer, second layer, and core; Figure S4). eGFP-positive cells in random images were quantified with STEPanizer software (Version 2b28-7, [42]) by randomly projecting a point grid on the images. The grid points falling on nontumor tissue areas were subtracted from the total number of grid points (100 grid points/random image) and defined as counts on tumor cells, i.e., total counts of tumor cells. The number of grid points falling on eGFP-positive (red-stained) tumor cells, i.e., total counts of eGFP-positive cells, were used to calculate the percentage of eGFP-positive cells in the tumor area.

### 2.8. Uncoating Assay from Tissue

The indirect determination of the efficiency of vector genome release (“uncoating”) was performed as previously described for in vitro cultured cells [43] with few changes. In detail, 900 ng of tissue DNA was diluted in 50 µL reaction volume and treated with T5 exonuclease (M0663, 30 Units, New England Biolabs, Frankfurt a.M., Germany) at 37 °C for 30 min, then incubated 10 min at 70 °C and diluted 2-fold to inactivate the enzyme. In parallel, 900 ng of the same DNA sample were mock-treated in the same reaction volume. Finally, 2-µL samples were subjected to qPCR quantification. The percentage of episomal DNA was calculated from the ratio of T5-resistant vector DNA to total vector DNA.

### 2.9. LDH Measurements

The release of LDH into the culture medium was determined as a marker of membrane integrity. Total lysis controls (TLCs) were prepared by adding 1% Triton^®^ X-100 to the medium of the control slices. Then TLCs were incubated for 1 h at 37 °C. Thereafter, slices were homogenized for 20 s in detergent-containing medium using an Ultra-Turrax^®^ T8 (IKA^®^-Werke, Staufen, Germany). After a centrifugation step, supernatants of both LDH and TLC samples were diluted to 1:10 with PBS. The LDH activity was then determined using a Cytotoxicity Detection Kit (Roche Diagnostics GmbH, Penzberg, Germany) and a SpectraMax^®^ 340 PC Microplate reader (Molecular Devices, San José, CA, USA) at 490 nm. LDH release is shown as a percentage of total lysis control.

### 2.10. Statistical Analysis

All statistical analyses were performed in GraphPad Prism 6 (GraphPad Software, Northside, San Diego, CA, USA). “*N*” is defined as the number of biologically independent experiments, whereas “*n*” describes the number of technical replicates. Data with *N* ≥ 3 biologically independent experiments/samples were subjected to statistical analysis.

The raw data and/or log_10_-transformed data were inspected visually and tested—if possible (*N* ≥ 7)—against the hypothesis of normal distribution using the Shapiro–Wilk test. For samples with *N* < 7, normal distribution was assumed. When no evidence against the hypothesis of normal distribution was found, a parametric two-tailed *t*-test for two-group comparisons was used. Additionally, a Welch correction was applied if the standard deviations (SD) of the two groups were significantly different. Multiple group comparisons were analyzed with an ordinary one-way ANOVA, followed by a Tukey’s post hoc test. When a control column was available (e.g., “Liver”), the remaining groups were tested against the control column and corrected with Dunnett’s test for multiple comparisons.

In the case of evidence for non-normally distributed data, nonparametric tests were used (the Kruskal–Wallis test, followed by a Dunn’s post hoc test, for multiple group comparisons and the Mann–Whitney test for two-group comparisons). Again, if a control group was available in a multigroup comparison, the remaining groups were tested against the control, followed by Dunn’s post hoc test.

Data with *N* = 1 were not subjected to statistical analysis.

Results with a *p*-value ≤ 0.05 were considered statistically significant.

## 3. Results

### 3.1. Enhanced Transgene Expression in HCC

**Figure 1:**
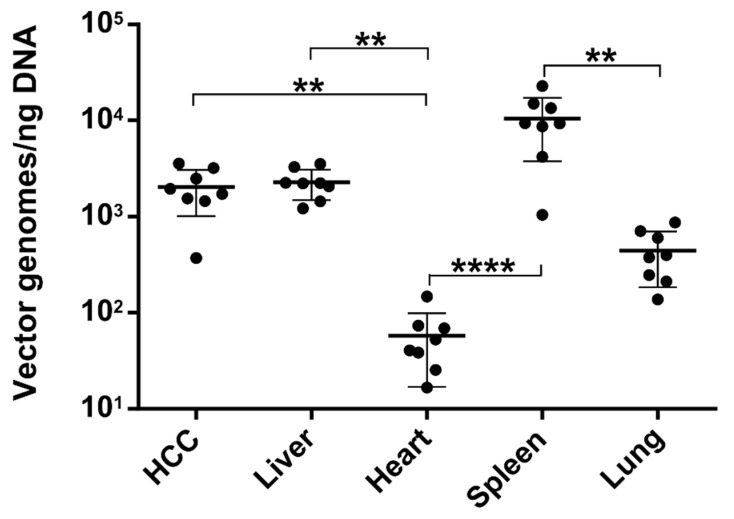
Biodistribution of AAV2 vector in the TGFα/c-myc HCC mouse model. Tumor-bearing animals injected intravenously with scAAV2.SFFV.Fluc and scAAV2.CMV.eGFP. Vector dose: 2 × 10^11^ particles per vector per animal. Relative vector genomes were quantified by qPCR 96 h after the first vector (scAAV2.SFFV.Fluc) administration. The levels of firefly luciferase transgene were normalized to ng DNA. Data are shown in log_10_-scale and as the mean target-to-reference ratio per animal with mean and SD. Statistics: Cohort sizes account for Shapiro–Wilk normality test (*p* ≤ 0.0459). Shapiro–Wilk normality test with log_10_-transformed data (*p* = 0.0209); Kruskal–Wallis test (*p* < 0.0001) and Dunn’s test ** *p* < 0.01, **** *p* < 0.0001). *N* = 8 animals.

**Figure 2:**
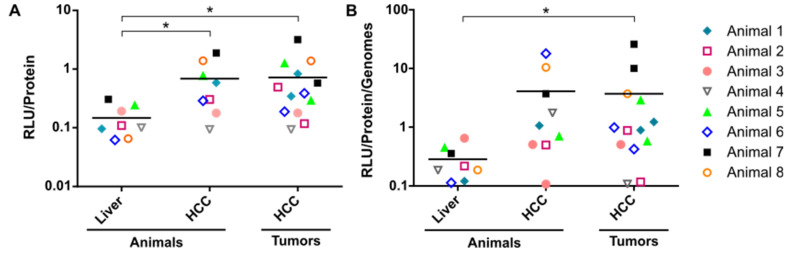
Transduction efficiency of AAV2 vector in liver and HCC of the TGFα/c-myc HCC mouse model. Tumor-bearing mice received scAAV2.SFFV.Fluc and scAAV2.CMV.eGFP. Vector dose: 2 × 10^11^ particles per vector per animal. (**A**) Transgene expression was analyzed by luciferase assay quantifying luciferase activity in liver and HCC 96 h after administration of the first vector (scAAV2.SFFV.Fluc). As outlined above, animals had been first i.v. injected with scAAV2.SFFV.Fluc and 12 h later with scAAV2.CMV.eGFP. Luciferase activity is shown in relative light units (RLU) normalized to the total protein content of tissue lysates in µg. (**B**) Transgene expression index determined by correlating RLU (normalized to protein content) and relative vector genomes (normalized to ng DNA; see Figure 1). Data for (**A**,**B**) are shown in log_10_-scale and as mean units per animal (liver and HCC tissue), as well as mean units per individual tumor sample (*n* = 1–2 tumor lesions per animal compared to respective liver sample), together with the grand mean within each cohort. Samples belonging to the same animal are labeled by the same symbol. Statistics: Cohort sizes account for the Shapiro–Wilk normality test (*p* < 0.0001); Shapiro–Wilk normality test with log_10_-transformed data (not significant (ns)); ordinary one-way ANOVA with log_10_-transformed data ((**A**): *p* = 0.0121; (**B**): *p* = 0.04) and Dunnett’s multiple comparison test with “Liver” as control cohort; * *p* < 0.05. *N* = 8 animals.

**Figure 3:**
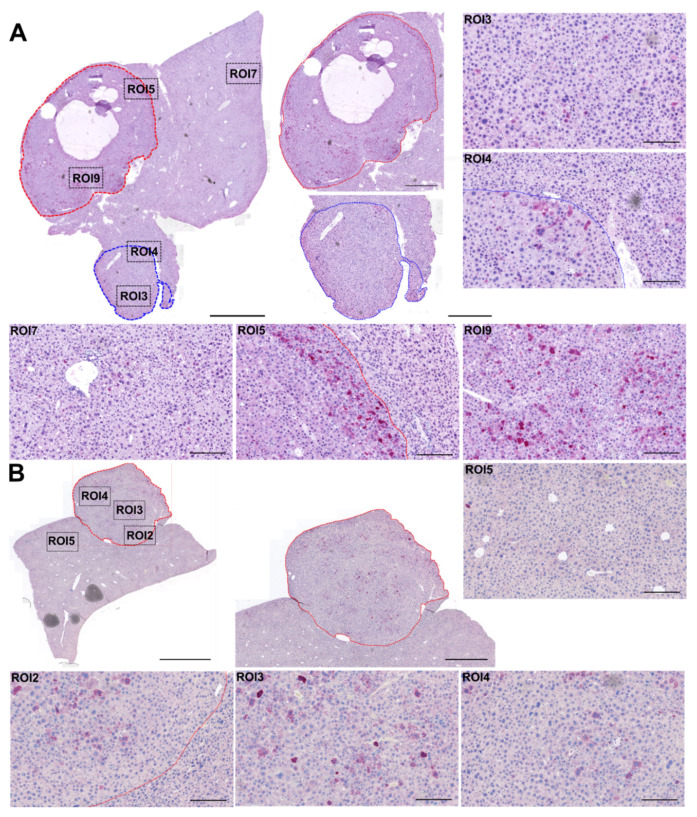
Immunohistochemical analysis of transgene (eGFP) expression in liver and HCC of TGFα/c-myc HCC mice. Tumor-bearing mice received scAAV2.SFFV.Fluc and scAAV2.CMV.eGFP. Vector dose: 2 × 10^11^ particles per vector per animal. Representative histological images of tumor-bearing liver sections of (**A**) Animal 2 and (**B**) Animal 8, 84 h post scAAV2.CMV.eGFP administration, which equals 96 h of scAAV2.SFFV.Fluc presence. eGFP-positive cells (in red) in the liver and HCC were visualized by immunostaining using Abcam anti-eGFP antibody. ROI = region of interest. Scale bars: total section in (**A**): 1 mm, in (**B**): 2 mm; magnified tumor lesions (**B**) (red frame): 1 mm; ROIs in (**A**,**B**) 0.2 mm. *N* = 8 animals.

**Figure 4:**
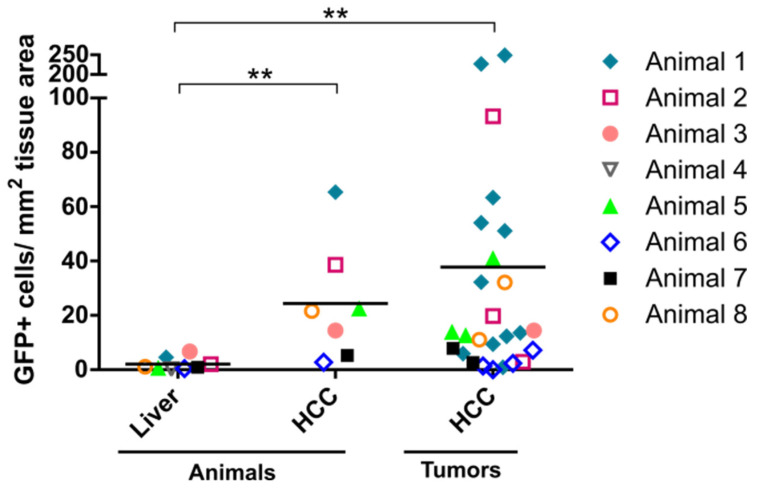
Transgene-expressing cells in liver and liver tumor tissue of TGFα/c-myc HCC mice. eGFP-positive cells in the liver and tumors were quantified per tissue area in mm^2^. Samples belonging to the same animal are labeled by the same symbol. Data are derived from the same animals as in Figure 1, Figure 2 and Figure 3 and are shown in linear scale as mean cells/mm^2^ per animal and mean cells/mm^2^ per individual tumor with grand mean within each cohort. Data are shown in linear scale. Statistics: Cohort sizes account for the Shapiro–Wilk normality test (*p* < 0.0001); Shapiro–Wilk normality test with log_10_-transformed data (*p* ≤ 0.0249). Kruskal–Wallis test (*p* = 0.002) and Dunn’s multiple comparisons test with “Liver” as control cohort (** *p* < 0.01). *N* (liver) = 8 animals; *N* (HCC) = 7 animals; *n* = 26 tumor lesions.

Tropism of AAV2—defined as cell-preferred transduction—was investigated in a TGFα/c-myc HCC mouse model. In this model, hepatocarcinogenesis is accelerated upon ZnCl_2_-mediated induction of TGFα and c-MYC oncogene overexpression [35]. The formation of endogenous tumors was monitored by magnetic resonance imaging. Tumor-bearing mice were treated with two different AAV vector constructs for comprehensive analyses, excluding at the same time possible transgene- or promoter-related effects. Specifically, we first injected mice with AAV2 vectors encoding for firefly luciferase (Fluc) controlled by spleen focus-forming virus (SFFV) promoter in a self-complementary (sc) vector genome conformation (scAAV2.SFFV.Fluc). Twelve hours later, AAV2 vector particles encoding for enhanced green fluorescent protein (eGFP) controlled by a cytomegalovirus (CMV) promoter (scAAV2.CMV.eGFP) also in sc conformation were administered. We chose the sc AAV vector genome conformation to exclude any bias due to second-strand synthesis. Vector distribution, as well as transgene expression, was determined 96 h after scAAV2.SFFV.Fluc administration (Figure 1, Figure 2, Figure 3 and Figure 4). Therefore, tumor nodules and nontumor liver tissues were collected, as well as heart, lung, and spleen.

We first focused on the luciferase-containing AAV2 vectors and determined their biodistribution by a quantitative polymerase chain reaction (qPCR). In line with previous reports [31,44], the highest levels of vector genomes (vg) were detected in the spleen (HCC: 1.0-fold SD ± 0.5; spleen: 5-fold SD ± 3; *p* > 0.7597) and liver. Interestingly, in liver and HCC, comparable levels of AAV vector genomes were detected (Figure 1; HCC: 1.0-fold SD ± 0.5; liver: 1.2 SD ± 0.4-fold; *p* > 0.9999)). In contrast, significantly lower levels were measured for the heart (approximately 35-fold SD ± 18 < HCC; (*p* = 0.0079) and a trend for reduced levels of vg for the lung (approximately 162-fold SD ± 82 < HCC; *p* = 0.5433) was observed.

Next, we analyzed transgene expression by measuring luciferase activity in lysates of livers and HCCs (Figure 2). Since most animals contained multiple tumors, values are presented as mean per animal and—alternatively—as mean per individual tumor. For both settings, a 5-fold (animals: SD ± 4; *p* = 0.0408, and individual tumors: SD ± 6 *p* = 0.0206) higher firefly luciferase activity was measured in HCC compared to liver lysates (Figure 2A). Differences were also obvious when correlating transgene expression and vector genomes. Specifically, transgene expression efficiency, defined as transgene expression per vg, was up to 13-fold (individual tumors: SD ± 25; *p* = 0.0352) higher in HCC compared to the liver (Figure 2B).

With regard to the second vector, delivering eGFP as a transgene, we determined the number of eGFP-positive cells in the liver and HCC. Therefore, we performed immunohistochemical analyses of HCC samples and adjacent liver tissues. Again, we observed clear differences in transduction between HCC and liver tissue (Figure 3 and Figure 4). Specifically, strong transgene expression was detected in the area next to the border and in the central area of the tumor lesions. In contrast, only moderate expression was detectable in adjacent nonmalignant liver regions (Figure 3).

When quantifying eGFP-positive cells, a higher number of eGFP-positive cells per mm^2^ tissue area—independent of whether depicted as mean per animal or mean of individual tumor samples—was observed in HCC (Figure 4). When comparing the values obtained for whole animals, approximately 13-fold (SD ± 10) (*p* = 0.0056) more eGFP-positive cells were present in HCC versus liver tissue. In line, when comparing individual tumor samples and the liver of the same animal, 19-fold (SD ± 30) (*p* = 0.0021) more eGFP-positive cells were determined. Based on these striking results, we set up a pilot study in the LT2/MYC model, which is also a transgenic MYC-driven HCC model. We confirmed the better performance of AAV2 vectors in HCC as compared to the liver in the LT2/MYC model (Figure S1).

In summary, AAV2 vectors accumulate in the liver and liver tumor tissue of transgenic HCC-bearing mice with comparable efficiency, whereas transgene expression levels were significantly higher in the tumors. This enhanced transduction efficiency was independent of the promoter and the transgene.

### 3.2. AAV2 Vector Transduces All Layers, including the Core of Tumor Lesions

To determine whether all tumor areas are accessible to intravenously administered vectors, we injected scAAV2.CMV.eGFP vectors into tumor-bearing TGFα/c-myc HCC mice and stained tumor sections for eGFP using an anti-eGFP antibody. Random images encompassing 30 percent of the total tumor area were generated (Figure S2).

**Figure 5:**
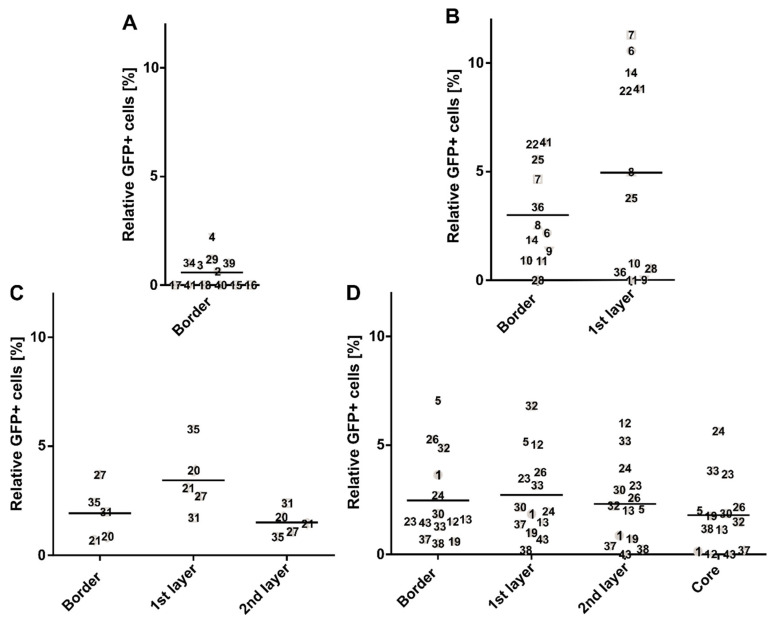
Transduction of all tumor layers by AAV2 vector in TGFα/c-myc HCC mice. Tumor-bearing animals were i.v.-injected with scAAV2.CMV.eGFP. Vector dose: 1 × 10^11^ particles per animal. Four days post-administration, HCC lesions were isolated, and transgene (eGFP) expression was analyzed from histological images. Enhanced GFP-positive cells in HCC were visualized by immunostaining. Enhanced GFP-positive cells per total tumor cells were quantified from random images of 30% of the tumor area of each lesion. Random images were assigned to distinct tumor layers defined as the border, the first layer, the second layer, and the core. (**A**) eGFP-positive cells in tumors comprising the border; *n* = 12. (**B**) eGFP-positive cells in tumors comprising the border and first layer; *n* = 12. (**C**) eGFP-positive cells of tumor lesions comprising the border, first and second layer; *n* = 5. (**D**) eGFP-positive cells of tumor lesions, which comprise all defined layers; *n* = 14. Data points are labeled by sample number of analyzed tumor, 1 to 43. Data are shown in log_10_-scale as the average radiance with the grand mean within each cohort. Statistics: Cohort sizes of (**B**,**D**) account for the Shapiro–Wilk normality test (*p* < 0.0001); Shapiro–Wilk normality test with log_10_-transformed data ((**B**) *p* = 0.0147; (**D**) *p* = 0.0427). (**A**) Mann–Whitney test (ns); (**B**) ordinary one-way ANOVA with log_10_-transformed data (ns) and Tukey’s multiple comparisons test (ns), (**C**,**D**) Kruskal–Wallis test (ns), and Dunn’s multiple comparisons test (ns).

As a measure of vector penetrance, we first defined distinct tumor layers, representing the border, the first and second layer, and the core of the tumor lesions (Figure S3). Next, we quantified the number of eGFP-positive cells in each layer. In total, 43 tumor lesions were analyzed (Figure 5). While we observed slightly higher numbers of eGFP positive cells in the first tumor layer, eGFP expression was detected in all analyzed layers, including the core (Figure 5). These results show that AAV2 can travel across the tumor tissue to tumor areas distant from the border zone and with moderate vascularization.

### 3.3. Confirmation of HCC Tropism in Transgenic LT2/RAS HCC Mice

**Figure 6:**
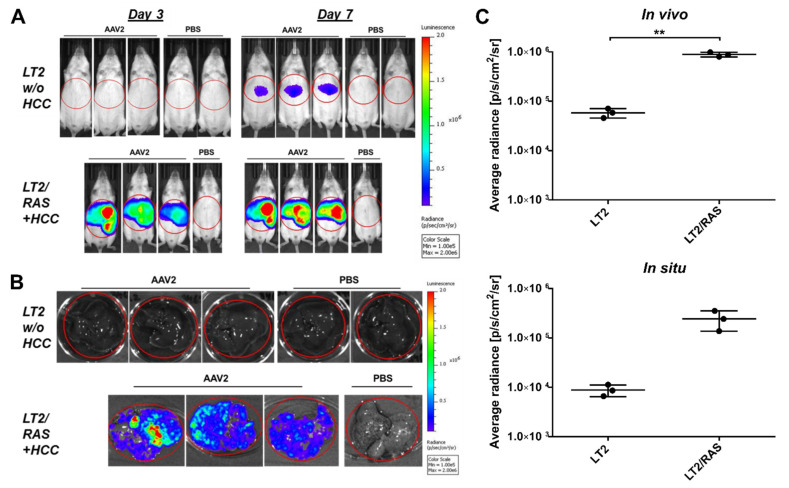
Transgene expression of AAV2 vector in liver and HCC in LT2/RAS HCC mice and controls. Indicated animals were i.v. injected with scAAV2.SFFV.Fluc and i.p. injected with D-luciferin 5 min before IVIS measurement. Vector dose: 1 × 10^11^ particles per animal. IVIS measurements of healthy LT2 mice compared to tumor-bearing LT2/RAS HCC mice showing the AAV2 vector-mediated luciferase activity (**A**) in vivo on days 3 and 7 after vector injection and (**B**) of isolated liver samples (in situ) on day 7 after vector injection. Luciferase activity quantified as average radiance (p/s/cm^2^/sr) of the luminescence signal, scale bars: Average radiance in p/s/cm^2^/sr, min = 1 × 10^5^, max = 2 × 10^6^; (**C**) IVIS quantification of average radiance in vivo and in situ on day 7. Data are shown in log_10_-scale as the mean average radiance with SD. Statistics: Unpaired two-tailed *t*-test with Welch’s correction (** *p* < 0.01). *N* = 3 animals with AAV treatment and *n* ≥ 1 with PBS treatment.

In the LT2/RAS HCC mouse model, HCC is caused by activated H-RASV12 [38,39], not by MYC unlike in our previous models. Tumors from LT2/RAS mice are composed of patches of tumor cells interspersed with nontumor regions spread across the liver parenchyma. This is in contrast to the defined tumor nodules developed in our MYC-induced mouse models (Figure S4). We, therefore, compared LT2/RAS mice that had developed HCCs to tumor-free LT2 mice. Mice were i.v. injected with scAAV2.SFFV.Fluc and luciferase activity was IVIS monitored on days 3 and 7 after vector administration (Figure 6A).

While, in healthy LT2 control mice, no luciferase activity was detectable, all three HCC-bearing mice showed a strong signal on day 3 after vector administration. Also, on day 7, our second time point, a clearly enhanced transgene expression efficiency was detected in the LT2/RAS HCC mice compared to the nontumor-bearing control mice (LT2). Specifically, luciferase activity was detectable only at background levels in LT2 mice, while a strong signal was observed in AAV2 vector-treated LT2/RAS HCC mice. Whole-body in vivo imaging results were confirmed by in situ IVIS measurements of livers isolated from the tumor-bearing (LT2/RAS), nontumor (LT2), and control mice (Figure 6B). Strong luciferase activity was detected in tumor lesion-bearing livers of LT2/RAS HCC mice, which had received scAAV2.SFFV.Fluc, while the livers of PBS-injected LT2/RAS HCC mice, as well as livers of all LT2 mice (vector or PBS treated), showed no luciferase activity. Quantification revealed a 15- (SD ± 2; *p* = 0.0078) to 28-fold (SD ± 12; *p* = 0.0632) higher activity in scAAV2.SFFV.Fluc-treated HCC-bearing livers compared with livers from LT2 mice (Figure 6C).

### 3.4. The Number of Episomal Vector Genomes Differs in HCC and the Liver

**Figure 7:**
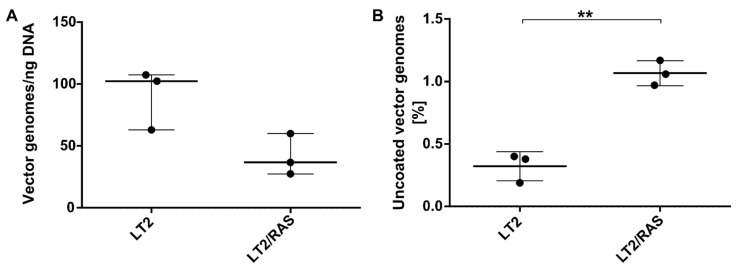
Quantification of T5-resistant episomal vector DNA as indication for uncoating efficiency in HCC and liver samples of AAV2 vector-treated LT2 and LT2/RAS mice. The number of AAV vector genomes in episomal form was compared between healthy liver tissue (LT2) and tumor-bearing liver tissue (LT2/RAS) seven days after vector administration. Samples were obtained from the animals shown in Figure 6. (**A**) Total and (**B**) uncoated vector genomes (episomal conformation) were quantified by qPCR in tissue DNA after mock treatment or T5 exonuclease treatment, respectively. Data are shown in linear scale and as (**A**) Firefly luciferase transgene copies normalized to ng DNA per reaction and (**B**) mean percentage of uncoated vector genomes in mock-treated tissue DNA. Statistics: (**A**) Unpaired two-tailed *t*-test with Welch’s correction (ns); (**B**) Unpaired two-tailed *t*-test (** *p* < 0.01). *N* = 3 animals.

Since we observed a comparable entry efficiency but a significant difference in transgene expression for AAV2 between HCC and liver, we hypothesized that the intracellular microenvironment of HCC might facilitate the release of vector genomes from capsids, a step known to limit transduction. Therefore, we performed an indirect uncoating assay on DNA isolated from HCC and liver samples of the LT2 and LT2/RAS mouse models. The assay makes use of the different conformations of encapsidated and released vector genomes, i.e., linear DNA versus episomes (concatemers), which show sensitivity or not to T5 exonuclease treatment, respectively [43]. The number of vector genomes was nonsignificantly lower in tumor-lesion-bearing LT2/RAS livers (Figure 7A). Interestingly, in liver tissues of LT2 mice, only 0.3% (SD + 0.1%) of AAV vector genomes were found to be T5 exonuclease resistant, whereas 1.1% (SD + 0.1%) were measured in tumor-bearing LT2/RAS liver tissues (Figure 7B), representing a 3.7-fold (*p* = 0.0011) increase in vector genomes in the episomal form, which can only be adapted once vector genomes are released from the capsid, in HCC compared to liver.

### 3.5. AAV2 Vector Preference for HCC Tissue Observed in Human Precision-Cut Liver Slices

**Figure 8:**
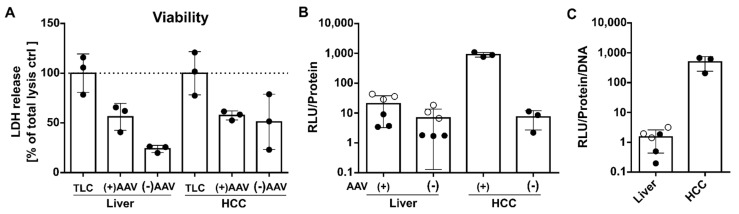
AAV2 expression in HCC compared to liver tissue in a human PCLS model. In vitro cultured PCLS processed from human liver parenchyma (P-PCLS) and HCC tissue (T-PCLS) were transduced with 4 × 10^10^ sc.AAV2. SFFV.Fluc vector particles per 2 PCLS (Ø 8 mm, 200 to 300 µm strength) per well; 24 h p.t., PCLS were harvested. (**A**) Viability analysis of HCC patient-derived PCLS by LDH assay. ODs are normalized to untransduced total lysis control (TLC), which was treated as “total death” control with Triton-X 100. Data are shown in the linear scale as the LDH release in % with mean and SD. (**B**) Luciferase activity is depicted as relative light units (RLU) normalized to protein content, quantified by Bradford assay. Data are shown in log_10_-scale as the RLU per protein with mean and SD. (**C**) Transgene expression index of AAV-treated PCLS, defined as the ratio of luciferase activity to vector genome content. Data are shown in log_10_-scale as RLU per protein per DNA with mean and SD. Statistics: N/A for the number of independent experiments. (**A**) *N* = 1 HCC patient P-PCLS (liver), and *N* = 1 matched T-PCLS (HCC); (**A**,**B**). *N* = 1 non-HCC patient P-PCLS (liver, clear circles); *N* = 1 HCC patient P-PCLS (dark circles); *N* = 1 matched T-PCLS (dark circles). (**A**–**C**) are technical replicates (*n* = 3), with the mean values of 2 PCLS samples/well.

Next, we investigated whether the preference of AAV2 for HCC compared to the liver is restricted to mouse models or whether it also applies to HCC from patients. To answer this question, we used viable precision-cut slices (PCLS) of human HCC and liver (Figure S5). For transduction, sc.AAV2.SFFV.Fluc were added to either parenchyma- (P-) or tumor-derived (T) PCLS (Figure 8A). Viability was monitored by LDH assay, and LDH levels were detected at a maximum of approximately 58% (SD ± 5%) for the untransduced total lysis control (TLC) and were observed to be elevated in the transduced P-PCLS compared to the untransduced counterpart (Figure 8A).

PCLS were harvested 24 h post transduction (p.t.) and analyzed. Similar to the data obtained for the mouse model, the firefly luciferase activity was superior in AAV2 vector-treated HCC T-PCLS compared to the liver P-PCLS. When analyzing the vector genome content of P-PCLS in comparison to T-PCLS, the human tumor tissue contained significantly fewer vector genomes than the human liver parenchyma (Figure S5), suggesting that AAV2 vectors enter or are internalized into the cells of healthy human liver with higher efficiency. Nonetheless, fewer vector genomes in tumor tissue resulted in higher transgene activity, as observed by a luciferase assay (Figure 8C). In detail, in the HCC patient-derived T-PCLS, AAV2 expression was 205-fold (SD ± 90) higher than in the matched P-PCLS and 26-fold (SD ± 5) higher than in the non-HCC patient-derived P-PCLS. When building the ratio of RLU per vector genome (=AAV2 expression index), the index was 585-fold (SD ± 300) higher in the HCC patient-derived T-PCLS than in the matched P-PCLS and 229-fold (SD ± 117) higher than in the non-HCC patient-derived P-PCLS. Although we could only test samples from a single healthy and a single HCC-bearing human donor so far, the results underline our hypothesis that AAV2 benefits from distinct conditions within the HCC tissue, which appears to promote their intracellular processing, thereby increasing the efficacy of transduction of HCC compared to liver tissue.

## 4. Discussion

In order to develop novel treatment options for patients with HCC, efficient, safe, and in vivo applicable vectors are needed. All these criteria are fulfilled by AAV vectors [9], which are derived from the nonpathogenic Dependoparvoviridae and are already in use in market-approved in vivo gene therapies. Here, we report that AAV2 vectors possess a strong tropism for HCC. We observed this preference for malignant over nonmalignant hepatocytes in various transgenic HCC mouse models, which reflect the natural cause of HCC development and the histopathology much better than the commonly used xenograft models. Relevance for human HCC was demonstrated by analyzing human patient samples that clearly confirmed AAV2′s HCC tropism. This preference in transduction is independent of the promoter or the transgene as well as the oncogene that serves as the tumor driver. Interestingly, it is not due to improved uptake, as comparable (Figure 1) or even lower vector genome levels (Figure S5) were detected in HCC compared to nonmalignant liver tissue. In contrast, intracellular conditions in HCC seem to promote the release of vector genomes from AAV capsids, a prerequisite for transgene expression that occurs less efficiently in nonmalignant liver tissue (Figure 7).

AAV vectors are considered the delivery tools of choice for liver-directed gene therapy. They present, so far, a favorable safety profile, can be purified to high titer, commonly show only mild and transient immune reactions, and are maintained as episomes in the cell nucleus of transduced cells. Although AAV2 vectors were the first to be vectorized [45] and the first to be tested for liver-directed gene therapy, the focus in pre-clinical development soon shifted towards other serotypes. In particular, AAV8 and AAV5 [46], for which the seroprevalence in the human population is low, and AAV3, which transduces liver cells using hHGFR [28], have gained attention. In addition, AAV6 [47], AAVrh10 [48], and AAV3B ST [30] have been reported to confer high transduction efficiencies in nontumor-bearing livers.

Here, we revisited AAV2 in the context of HCC and made the interesting observation that, in the same tissue, malignant cells are clearly preferred over nonmalignant cells concerning transduction. AAV2 uses heparan sulfate proteoglycan (HSPG) as a primary attachment receptor [49]. The negatively charged polysaccharide chain of heparan sulfate, which interacts with distinct residues of the capsid (R484, R487, K532, R585, and R588) and which is expressed on hepatocytes, is discussed as a host factor determining the liver tropism of AAV2 [50,51,52]. The HSPG density and glycosylation pattern on liver cancer cells are altered [53] and may be involved in enhanced intracellular processing as HSPG is suspected to not only serve as an attachment receptor but also to affect transduction processes. Moreover, AAV2, as vectorized in laboratories, is reported to be less efficient at hepatocyte transduction due to the relatively high HSPG affinity [51,52]. A further receptor of AAV2 that might play a role is HGFR (syn. c-Met, [54]), given the explicit dependency of AAV3 on hHGFR for HCC and hepatocyte transduction. hHGFR shows 88% homology to its murine counterpart. However, variations are found in the extracellular domain, where interactions with viruses are expected [28]. Interestingly, in contrast to the strong preference for human HGFR reported for AAV3, AAV2 transduces murine and human hepatocytes and HCCs. HGF-HGFR signaling is essential for hepatocyte proliferation and survival and is upregulated in HCC [29]. In addition, HSPG-dependent accumulation of HGF and clathrin-dependent HGFR internalization and intracellular trafficking to the Golgi apparatus resemble AAV’s infectious pathway [54] and may enhance AAVs transduction efficiency. Furthermore, like for most AAV serotypes, intracellular processing of AAV2 depends on AAV receptor (AAVR) [55] and GPR108 [56], host factors that are guiding the AAV particles through the endosomal compartment towards the trans-Golgi network. On this route, the N-terminus of AAV’s major capsid proteins VP1 and VP2 become externalized. These and other changes to the capsid are required to prime vector uncoating, a step in the vector–host interaction that in some cell types such as dendritic cells poses a barrier to transduction [44]. Our finding of lower numbers of vector genomes in the episomal/concatemeric conformation, and thus clearly released from the AAV capsid in hepatocytes vs. HCC (Figure 7), is in line with a report by Thomas and colleagues demonstrating that uncoating efficiency represents a limiting factor in murine hepatocyte transduction for AAV2 compared to AAV8 and AAV6 [57]. Furthermore, Miao et al. pointed to a correlation between episomal AAV vector DNA levels and transgene expression in hepatocytes [58]. Therefore, we suggest that AAV2 uncoating occurs in hepatocytes with low efficiency, while conditions in HCC are more favorable. However, uncoating efficiency may be one of several potential factors contributing to AAV2′s enhanced transduction efficiency in HCC.

Furthermore, as a more general finding, which is again in line with our results, a remarkable discrepancy between vector-containing and transgene-expressing hepatocytes has been observed by different groups following systemic AAV vector application in mice. Specifically, these studies report up to 100% of vector DNA-positive murine hepatocytes, while only 5–10% of murine hepatocytes show transgene expression, even at high vector doses [58,59,60,61,62,63,64,65]. The same holds true for human hepatocytes as transduction efficiency can be greatly enhanced by changing the intracellular microenvironment, i.e., following the uptake of vectors into cells. For example, by inducing autophagy [66] or adding supporting factors such as HBx of hepatitis virus B [67], the delivery of AAV vector particles to the nuclei of hepatocytes and, in particular, transgene expression was significantly enhanced. While the nature of the barrier(s) remains to be elucidated, the enhanced permissiveness of HCC for AAV2 discovered in the present study may represent a feature that allows for the development of novel strategies for AAV vector-based treatment for HCC.

AAV2 vectors have been studied exclusively in grafted mouse models (subcutaneous and orthotopic; syngenic and xenografts) [11,12,13,14,15,16,19,26]. Vectors were administered intravenously, intratumorally, or via a portal vein injection. Alternatively, hepatoma cell lines were treated ex vivo, followed by transplantation. These are highly artificial systems that do not allow for the study of the natural interaction of AAV with the malignant and nonmalignant cell types of the tumor tissue. Furthermore, xenografted models are based on immunodeficient mouse models, which do not reflect natural immunological barriers to AAV. Therefore, we focused on tumor models relying on endogenous tumorigenesis. To our knowledge, ours is the first comprehensive analysis of AAV2 in transgenic HCC mouse models. There are reports on using AAV8 vectors in transgenic HCC, but none of these studies investigated HCC vs. parenchymal liver transduction [17,23,25,68] or reported on significant differences in transduction between nonmalignant liver parenchyma and tumor tissue [39], which points to AAV2-specific barriers to hepatocyte transduction, which are absent or overcome in HCC cells.

When analyzing AAV2 in transgenic mice with endogenously formed HCCs, we observed that AAV2 vectors possess an intrinsic, i.e., natural, preference for HCC transduction. In the TGFα/c-myc model, tumor lesions and liver tissues contained similar amounts of AAV vector genomes, arguing against a preferred accumulation in tumor lesions due to morphological changes of the organ upon tumorigenicity. The same holds true for the LT2/RAS HCC mouse model, in which tumor lesions are spread across the whole liver parenchyma. Furthermore, the uniquely comprehensive histological analysis of the tumor tissue revealed that all layers of tumor lesions of the TGFα/c-myc model are reached, even in larger tumors comprising a border, first layer, second layer, and core. The preference of AAV2 for HCC transduction was dependent neither on the promoter nor the transgene since the change from SFFV promoter to CMV promoter and from firefly luciferase to eGFP as transgene resulted in similar observations. Further, AAV2′s HCC tropism is also independent of the microenvironment specifically present in TGFα/c-myc mice since we observed the same tropism in the LT2/RAS HCC mouse model, where hepatocarcinogenesis is driven by activation of H-RASV12 [38].

The strong preference of AAV2 for HCC compared to nonmalignant liver cells is an excellent basis for the development of novel AAV-based strategies for the treatment of HCC. If required, this intrinsic feature can be further enhanced by applying transcriptional or post-transcriptional targeting strategies and by further improvement of the intracellular processing of AAV vectors in HCC. For the latter, basic research on AAV vector biology is key, emphasizing cell type- or disease-specific barriers such as differences in the pH of endosomes, the presence of distinct innate immune sensors, or the activity of proteasomes or DNA repair components. With regard to targeting, post-transcriptional and transcriptional targeting strategies have shown promise in avoiding expression in off-target cell types, including antigen-presenting cells, in significantly increasing the level of transgene expression, and in protecting nonmalignant cell types from treatment-related toxicity. For example, the application of natural tissue-specific promoters, like the albumin promoter combined with alpha-fetoprotein (AFP) [11] or telomerase reverse transcriptase (TERT) [19] promoter, restrict vector genome expression to the oncogene-overexpressing tumor cells, while the use of optimized synthetic promoters finetunes the expression efficiency [69]. In addition, the incorporation of micro (mi)RNA target binding sites in the vector genome, like the insertion of miR122-binding-site tandems, prevents transgene expression in healthy liver tissue. At the same time, the hepatocarcinogenic downregulation of miR122 common in HCC will result in unaffected expression from AAV vector genomes [70].

## 5. Conclusions

In the present study, we have demonstrated the clear preference of AAV2 for transduction of HCC compared to nonmalignant liver parenchyma. Although we are aware that our results for patient samples await statistically robust verification through additional donor samples, the current results strongly argue that AAV2 is also an HCC-tropic serotype in humans—a feature that should be exploited to optimize the efficacy of gene therapy-based strategies to treat HCC, the sixth most common cancer worldwide.

## Data Availability

The data presented in this study are available on request from the corresponding author.

## Supplementary Materials

The following are available online at https://www.mdpi.com/article/10.3390/cancers14020427/s1, Figure S1: Transgene expression in LT2/MYC HCC mouse model and healthy control, Figure S2: Random imaging of tumor nodules, Figure S3: Representative example of tumor segmentation, Figure S4: The phenotype of tumor lesions in the (A) TGFα/c-myc and (B) LT2/RAS HCC mouse models, Figure S5: AAV2 vector genomes in HCC compared to liver tissue in a human PCLS model, Table S1: Nucleotide sequences of qPCR primer sets applied for absolute and relative quantification of AAV vector genomes in AAV vector preparations and tissue DNA.
