# Supplementary material for: Hepatocellular Carcinoma Is a Natural Target for Adeno-Associated Virus (AAV) 2 Vectors
Source: Cancers (Basel). 2022 Jan 15;14(2):427. doi: 10.3390/cancers14020427 (PMC8774135; doi:10.3390/cancers14020427)
Supplement: Supplementary file 1 [file cancers-14-00427-s001.zip › cancers-1494987-SI.pdf]

Supplementary figures

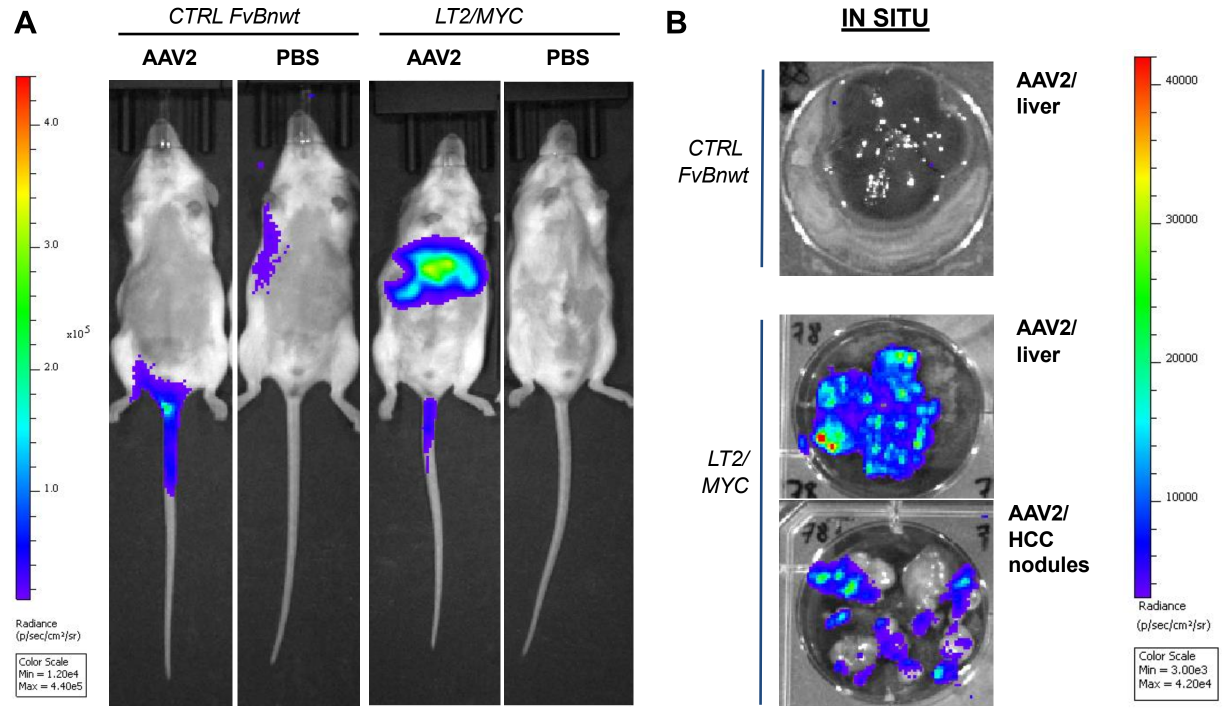

Figure S1. Transgene expression in LT2/MYC HCC mouse model and healthy control. Healthy control animals (CTRL FVB/N) and tumor-bearing animals (LT2/MYC) i.v.-injected with scAAV2.SFFV.Fluc (AAV2) or without AAV treatment (NC). Vector dose: 2×10^{11} particles per animal (A) In vivo imaging of luciferase activity 8 days post-transduction. (B) In situ imaging of luciferase activity in the healthy livers and tumor-bearing livers derived from AAV2 vector-treated mice shown in (A). Luciferase activity was detected as average radiance [p/s/cm²/sr].; *N* = 1 animal.

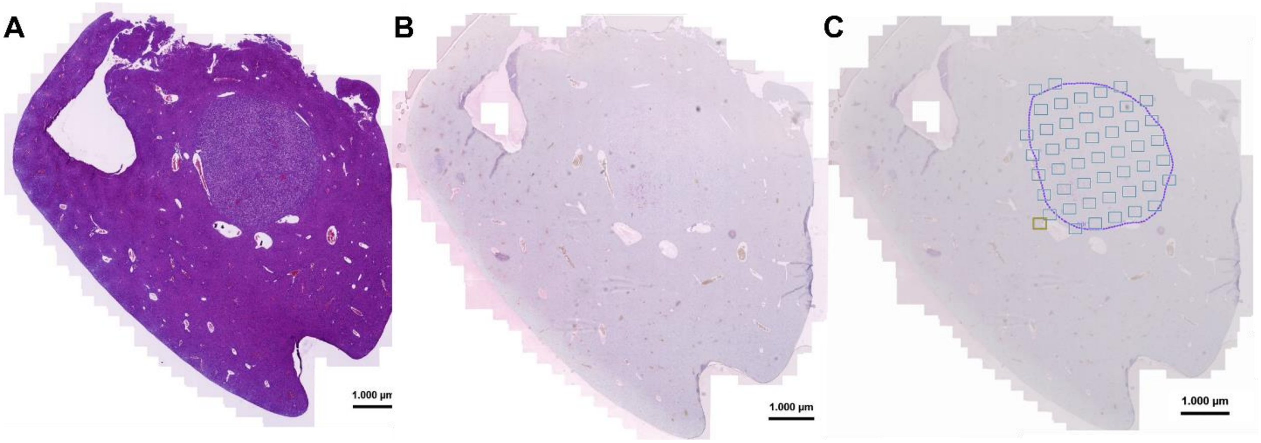

Figure S2 – Random imaging of tumor nodules. Representative images (AxioScan.Z1 slide scanner, Zeiss, Heidelberg, Germany) of (A) H&E- and (B) anti-GFP-stained tumor section scans, and of (C) random imaging (Visiopharm, Hoersholm, Denmark). The tumor lesion was marked as ROI, and random images were generated covering 30 % of the ROI area.

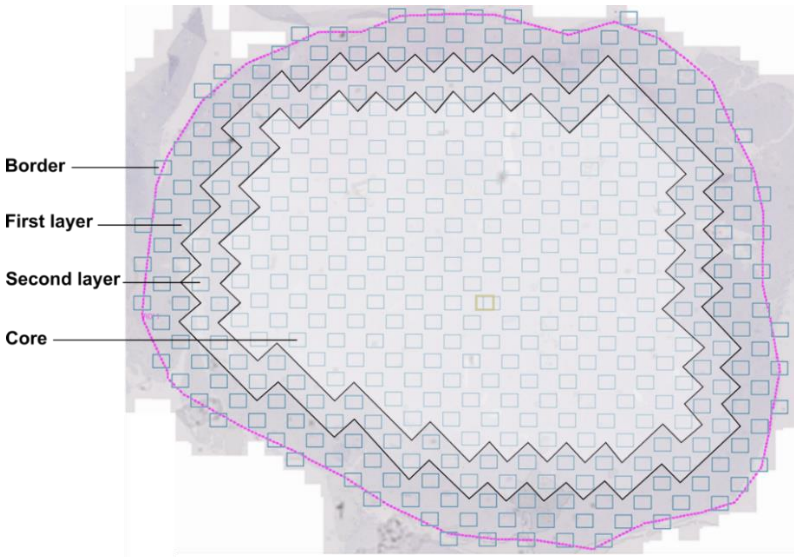

Figure S3 – Representative example of tumor segmentation: Tumor sections were segmented by defining the border (includes grids on the pink tumor outline), the first layer, the second layer, and the core. Segmentation was based on automatically generated random image grids (Visiopharm Software, 30 % of tumor area). Areas visualized with Inkscape.

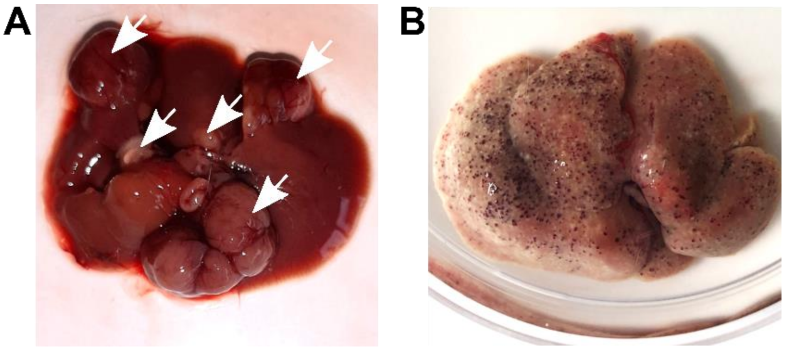

Figure S4. The phenotype of tumor lesions in the (A) TGFα/c-myc and (B) LT2/RAS HCC mouse models. Representative images of isolated livers with tumor lesions. (A) TGFα/c-myc-induced tumor lesions are endogenously formed as single to multiple defined, generally encapsulated HCC nodules. Arrowheads: tumor lesions (B) LT2/RAS-induced endogenous tumor lesions are spread across the whole liver parenchyma and not confined to nodules.

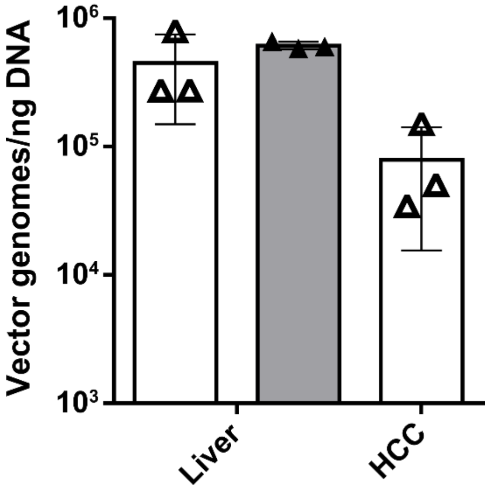

Figure S5 – AAV2 vector genomes in HCC compared to liver tissue in a human PCLS model. PCLS processed from human liver parenchyma (P-PCLS) and HCC tissue (T-PCLS) were transduced with 4×10^{10} sc.AAV2. SFFV.Fluc vector particles per 2 PCLS (Ø 8 mm, 200 to 300 μm strength) per well. After 24 h p.t., PCLS were harvested for luciferase assay and vector genome quantification. Vector genomes were quantified by qPCR with Fluc as target normalized to ng DNA per reaction. Data are shown in linear scale as Fluc-to-DNA ratio with mean and SD. *N* = 1 non-HCC patient P-PCLS (liver) (clear circles); *N* = 1 HCC patient P-PCLS (dark circles); *N* = 1 matched T-PCLS (dark circles); shown are technical replicates n = 3 mean values of 2 PCLS samples/well. Statistics: N/A for the number of independent experiments.

Supplementary tables

Table S1: Nucleotide sequences of qPCR primer sets applied for absolute and relative quantification of AAV vector genomes in AAV vector preparations and tissue DNA.
Fluc: Firefly luciferase

Primer	Sequence
Fluc.fw	5′ - TTC GGC TGG CAG AAG CTA TG – 3′
Fluc.rev	5′ - GCT CGC GCT CGT TGT AGA TG – 3′
GFP.fw	5′ – GCT ACC CCG ACC ACA TGA AG – 3′
GFP.rev	5′ – GCT CAT GCC GAG AGT GAT CC – 3′
